# Visual perceptual learning is effective in the illusory far but not in the near space

**DOI:** 10.3758/s13423-023-02389-w

**Published:** 2023-11-06

**Authors:** Antonio Zafarana, Alessandro Farnè, Luigi Tamè

**Affiliations:** 1 School of Psychology, University of Kent, Canterbury, CT2 7NP UK; 2 Lyon Neuroscience Research Centre, Impact Team, INSERM U1028, CNRS UMR5292, University Claude Bernard Lyon I, Lyon, France

**Keywords:** Perceptual learning, Visual learning, Peripersonal space, Extrapersonal space

## Abstract

Visual shape discrimination is faster for objects close to the body, in the peripersonal space (PPS), compared with objects far from the body. Visual processing enhancement in PPS occurs also when perceived depth is based on 2D pictorial cues. This advantage has been observed from relatively low-level (detection, size, orientation) to high-level visual features (face processing). While multisensory association also displays proximal advantages, whether PPS influences visual perceptual learning remains unclear. Here, we investigated whether perceptual learning effects vary according to the distance of visual stimuli (near or far) from the observer, illusorily induced by leveraging the Ponzo illusion. Participants performed a visual search task in which they reported whether a specific target object orientation (e.g., triangle pointing downward) was present among distractors. Performance was assessed before and after practicing the visual search task (30 minutes/day for 5 days) at either the close (near group) or far (far group) distance. Results showed that participants that performed the training in the near space did not improve. By contrast, participants that performed the training in the far space showed an improvement in the visual search task in both the far and near spaces. We suggest that such improvement following the far training is due to a greater deployment of attention in the far space, which could make the learning more effective and generalize across spaces.

## Introduction

It is well-established that sensory experience can change perceptual processes (Maniglia & Seitz, 2018; Seitz & Watanabe, 2005). This phenomenon is called perceptual learning, and it has been studied extensively by looking at several stimulus features, such as orientation (Schiltz et al., 1999), motion (Matthews & Welch, 1997), contrast (Sowden et al., 2002), texture (Karni & Sagi, 1991), and many others (Seitz, 2017). Visual studies have consistently demonstrated that performance can improve considerably after a certain amount of training (Fine & Jacobs, 2002; Watanabe & Sasaki, 2015). In a seminal study, Sigman and Gilbert (2000) tested participants on a visual search task in which they had to report whether a triangle with a specific orientation (0, 90, 180, 270 degrees) was present or not amongst 23 distractor triangles with different orientations arranged in a square. Then, they trained participants for a specific triangle orientation for 4–6 days. When participants were tested again on all the triangle orientations, their performance showed that visual perceptual learning occurred, specific to the orientation of the triangle. Namely, when the training was performed based on a particular stimulus orientation (e.g., triangle oriented upward), the performance improvement was specific to that orientation (e.g., triangle oriented upward). Furthermore, the learning effect was also restrained to the spatial location of the visual field in which the training was performed; thus, improvement was observed only at the specific eccentricity in which the training was performed and not at other eccentricities.

Space is a critical factor that influences perception of a stimulus position not only in azimuth and elevation but also in the distance dimension. There are different spaces in which stimuli can be coded: personal space (body surface), peripersonal space (PPS), and extrapersonal space (EPS). The PPS is a multisensory processing region surrounding our body, in which we interact with the close environment (Brozzoli et al., 2014; Bufacchi & Iannetti, 2018; Serino, 2019). The division of space in PPS and EPS was first suggested by studies in neglect patients who showed a selective impairment in the near and far spaces (Paterson & Zangwill, 1944). Putative neural underpinnings of PPS and EPS dissociation have been found in monkeys, which have revealed the existence of bimodal neurons in several brain areas (i.e., ventral intraparietal area, area 7b, ventral premotor cortex, and putamen) which are active when either visual or somatosensory inputs are present (Rizzolatti et al., 1981). Human analogue has been reported in neuroimaging studies (Brozzoli et al., 2011, 2013; Cléry et al., 2015; for review, see Brozzoli et al., 2014). However, these neurons fire more strongly when stimuli are close compared with far from the body (Graziano & Gross, 1993; Matelli et al., 1986; Rizzolatti et al., 1981). Similar neural structures were suggested to exist also in humans from both neuroimaging (Bernasconi et al., 2018; Brozzoli et al., 2011; Makin et al., 2007) and behavioural studies (di Pellegrino et al., 1997; Farnè & Làdavas, 2000; Halligan & Marshall, 1991; Serino et al., 2015).

Virtual reality (VR) and the Ponzo illusion are typically used to create a visual setting in which the space can be divided into two sections—one considered near (peripersonal) and the other regarded as far (extrapersonal; Ahsan et al., 2021; Blini et al., 2018). The “Ponzo illusion” (Ponzo, 1910) is an optical effect that leads the brain to misperceive the actual size of an object when it is presented in a perspective scenario (Gregory, 1963; Leibowitz et al., 1969; Prinzmetal et al., 2001). As a result, stimuli that have the same characteristics in terms of size are perceived and processed as different when placed in a perspective setting (distant stimuli are perceived as larger than near stimuli even if they have the same physical and retinal size). The brain uses depth cues to estimate the distance between the stimuli and their size is rescaled based on how far they seem to be by following Euclid’s law (Sperandio & Chouinard, 2015). However, recent studies have shown that the Ponzo illusion may not be explained by perceived depth features, as prior information and prediction errors may provide alternative explanations for the illusion (for a recent review on the topic, see Yildiz et al., 2022). Thus, this method allows the creation of a PPS and an EPS while maintaining the same physical visual angle for both spaces.

Recently, Blini et al. (2018), used a 2D Ponzo illusion and 3D VR settings to investigate human visual discriminative abilities in PPS and EPS spaces. They carried out a series of experiments in which they manipulated the retinal size (constant or naturally scaled) and the setting (Ponzo illusion or VR). They found that discrimination of objects located in peripersonal space was better than in extrapersonal space in terms of reaction time, with no speed/accuracy trade-off. Importantly, the results were similar across 3D and 2D settings (virtual reality and Ponzo illusion). Similarly, Ahsan et al., 2021; see also Dureux et al, 2021), explored whether perceived depth modulates performance on different visual tasks involving either low-level (size and orientation discrimination) or higher-level (face identification) visual properties using the Ponzo illusion. They showed that both precisions and reaction times were better when stimuli were presented in the near (PPS) compared with the far (EPS) space. These studies converge in showing that there is a general advantage for processing stimuli that are near compared with far from the body. Yet, whether this PPS processing advantage applies to visual perceptual learning remains unsettled.

Once established that there is better perception (i.e., both faster and more accurate visual object discrimination) in PPS than EPS, it can indeed be argued that the mechanisms subserving such a better perception may be at play also during visual training in the PPS, possibly leading to a spatially selective advantage in learning as well. In addition, recent empirical evidence indicates that another type of learning process—namely, associative learning—also displays spatial selectivity for the PPS. Using a Pavlovian fear-learning paradigm, Zanini et al. (2021) observed that fear responses were present only for visual stimuli within the PPS, indicating that the threatening valence of a visual stimulus is also learned according to its spatial proximity to the body (Zanini et al., 2021). Together, this evidence strengthens the grounds for the hypothesis of better visual perceptual learning in PPS than EPS.

Thus, here we investigated whether visual perceptual learning is affected by the location of the stimuli in space (near and far from the observer). Participants carried out a visual search task, both in the near space (PPS) and far space (EPS), whereby they had to report the presence of a target, which was a triangle with a specific orientation (either 0, 90, 180, or 270 degrees), randomly appearing amongst distractors bearing other orientations (modified after Sigman & Gilbert, 2000). Performance was assessed both before and after a training session in which participants were trained by repeated blocks of the visual search task, either in PPS or EPS, looking only for one specific orientation. Based on the aforementioned evidence (Ahsan et al., 2021; Blini et al., 2018; Zanini et al., 2021), we predicted to observe a larger improvement in performance, for the trained orientation (Sigman & Gilbert, 2000), in the group of participants who trained in the near compared with the far space. Moreover, owing to the previously documented degree of topological specificity of the improvement (Sigman & Gilbert, 2000), we predicted that the benefit following the near-space training would be specific for the PPS.

## Materials and methods

### Participants

Thirty-six participants (mean age = 28.17 years, *SD* = 7.86, range: 21–57; 23 females) took part in the study. We conducted a priori power analysis in G*Power (Version 3.1.9.7; Faul et al., 2009) using the data from (Sigman & Gilbert, 2000; *N* = 4) to estimate the minimum sample size to compare the performance for trained orientation before and after training. The effect size in Sigman and Gilbert’s (2000) study was 12.12, thus by setting an alpha criterion to 0.05 and power to 0.99, the resulting sample size for a two-tailed paired t-test was 3. Since they trained participants until they reached a certain performance level for the trained orientation, we calculated the effect size from the difference between trained and untrained orientations after training. For the same reason and because the number of trials in our study was substantially smaller (reduced to one fourth) and we had two different groups as well, we decided to use a bigger sample size (*N* = 18 each group). All participants had a normal or corrected-to-normal vision. The study was approved by the ethics committee of the School of Psychology, University of Kent, and was carried out according to the principles of the 1964 Declaration of Helsinki as updated by World Medical Association (2013).

### Apparatus and stimuli

**Fig. 1:**
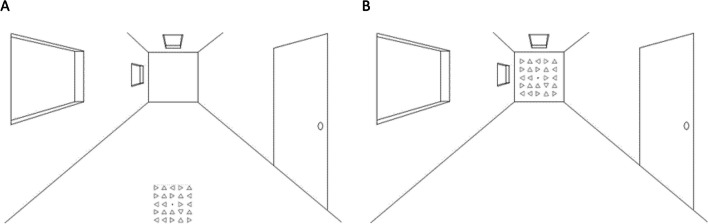
Depiction of the stimuli presentation in the near (**A**) and far (**B**) conditions. The stimuli were presented within a background scenario of a room. Note that the array of triangles in Panels **A** and **B** are of the same physical size

The visual search task was built in PsychoPy 3 (Peirce et al., 2019) and it was delivered online on Pavlovia. Participants were instructed to perform a series of online visual search tasks using their personal computers or laptops at home. The choice to perform the task online was primarily dictated by the COVID-19 outbreak at the time of the testing. Given that each participant used their own screen with different dimensions and resolution, the size of the visual stimuli was scaled based on the dimension of the participant’s display so that stimulus size remained constant across participants. To do so, participants had to resize a credit card appearing on their screen using the arrows on their keyboards. They used their credit card (which has a standard size) and positioned it on their screen superimposing it to the image. As mentioned, this process ensured a constant dimension of visual stimuli presented across the different participants’ screens. Although participants were instructed to position themselves at 90 cm from the screen, their actual distance could not be verified (online) and this may have added some degree of variability. Nevertheless, the relative distance between the near and far spaces remained constant within each participant, therefore, we believe that this variability did not affect the critical comparisons in our results. Visual stimuli consisted of 24 triangles arranged into a 5 × 5 matrix with a fixation dot in the middle (Fig. 1). Each triangle had black outlines and a white fill. The sides of every triangle were 7 mm in length and 6.1 mm in height, and they were located at 14-mm distance from their respective centres of mass. Therefore, the square matrix subtended 70 × 70 mm. Triangles were presented on top of a white background image depicting a room (see Fig. 1) which created a 2D depth perspective (Ponzo illusion), thus producing two illusory spaces (i.e., one near and another far from the observer). The near space was located in the lower half of the screen (Fig. 1A), whereas the far space was located in the upper half of the screen (Fig. 1B). Participants responded to the target presence by pressing P (target present) or A (target absent) on a keyboard (standard QWERTY keyboard).

### Design

**Fig. 2:**
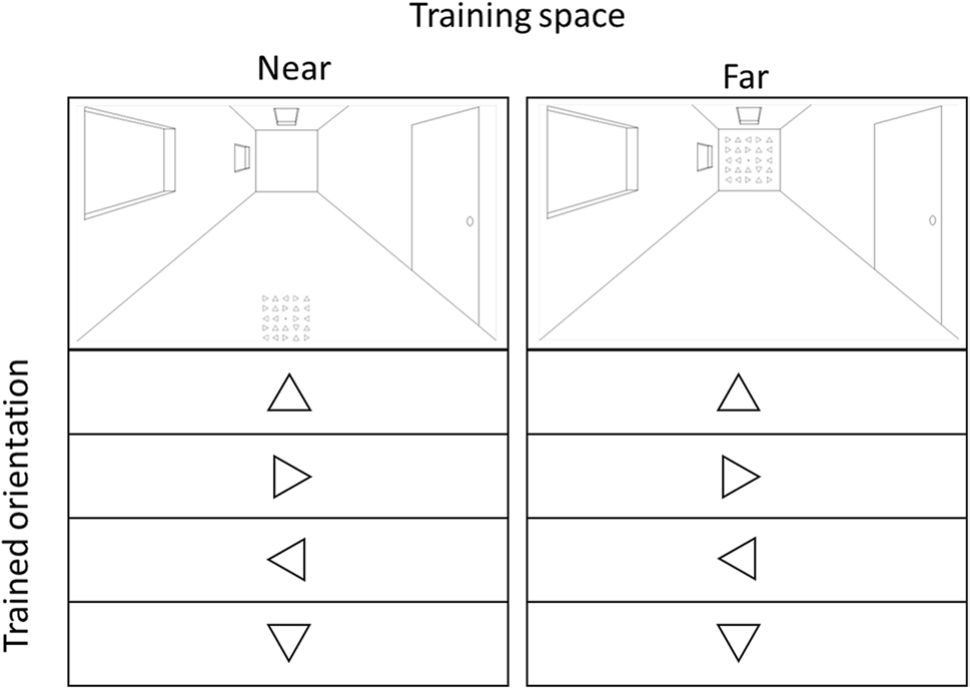
All possible experimental conditions. Participants were trained on one specific orientation (i.e., triangle pointing up, right, left, or down) either in the near or far space

There were four experimental conditions (see Fig. 2). Eighteen participants performed a training in the near space and another 18 in the far space. Moreover, the training was performed only for one of the four triangle orientations, the specific orientation varied across participants.

**Fig. 3:**
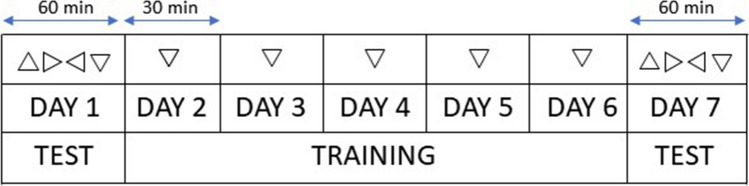
Timeline of the experiment including a first-day testing phase (Before training) lasting 60 minutes in which the four different triangle orientations were tested as targets. A five days training lasting 30 minutes in which only one orientation was used as a target. Note that the training orientation was varying across participants. Finally, the last day after-training testing phase in which all four orientations were tested as targets

The experiment consisted of two testing phases and a training period between the two phases (see Fig. 3). There were three within-participants factors: ORIENTATION (Trained, Untrained), TIME (Before, After), and SPACE (Near, Far) and one between-participants factor: TRAINING (in the near space, in the far space). The trained orientation was counterbalanced across participants.

### Procedure

Participants were contacted by email and received instructions on how to carry out the different tasks. The experiment lasted 7 days. Participants received one link for each day and used the links to access Pavlovia, which let them perform the tasks for the respective day. Participants received detailed instructions on how to carry out the tasks in a bullet list for each day. The experiment began with a testing phase, based on the sequence order (A: near-far, B: far-near), in which participants started the visual search task on the near or far space and then completed the task on the other space. The near and far blocks were performed separately, and the order was counterbalanced across participants. In this pretraining testing phase, participants were presented with a target, which was a triangle pointing either up, down, left, or right, at the beginning of each block (four in total, one for each orientation). Each block consisted of 150 trials, 20% of which were null (the target being absent). The target was presented 5 times in each of the 24 possible locations in the 5 × 5 matrix. There were a total of 1,200 trials, 600 in the near space and 600 in the far space.

**Fig. 4:**
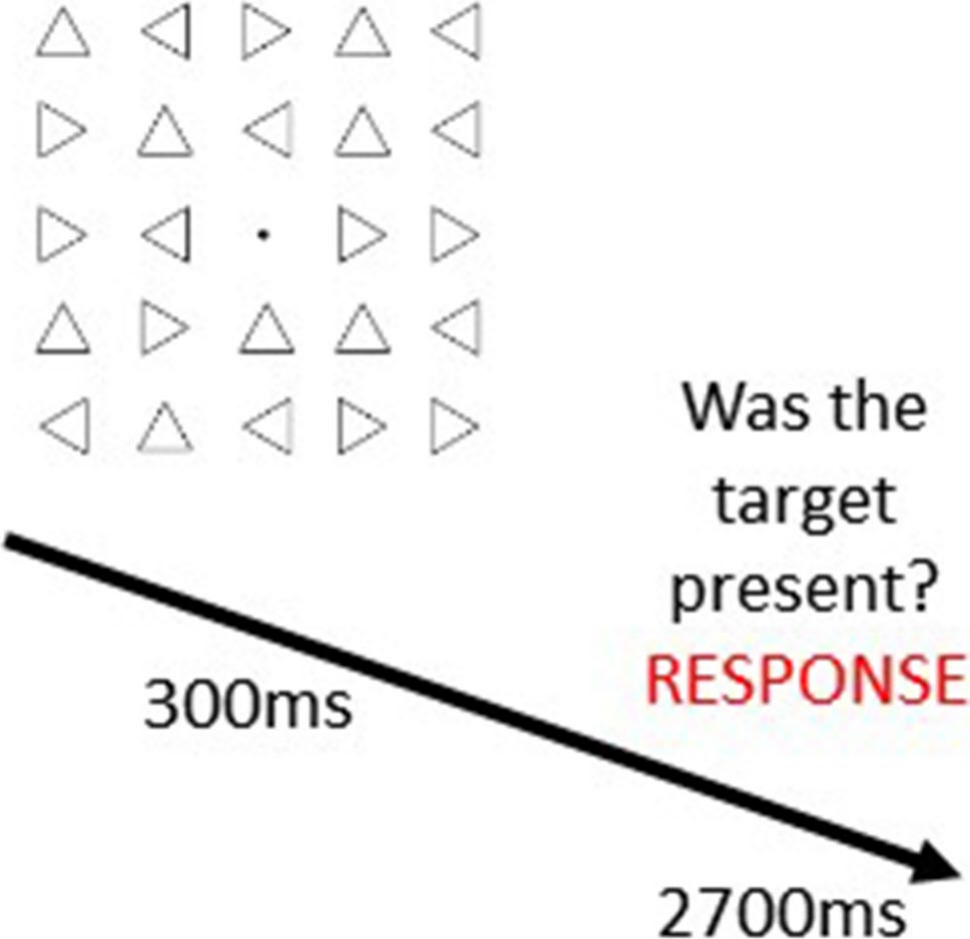
Visual search display example. At the beginning of each block, participants saw a target (e.g., a triangle pointing down) at the centre of the screen until they pressed the arrow on the keyboard pointing in the same direction to confirm they understood the orientation. In each trial, 24 triangles appeared for 300 ms. Immediately after participants had to report whether the target was present or absent. The triangles seen above here were presented in the near or far space

Each trial had a total duration of 3,000 ms and started with the presentation of the visual stimuli for 300 ms (Fig. 4). Then, participants had 2,700 ms to give their response before the start of the next trial. The subsequent stimulus was presented even if the participant did not report any response. The background image (i.e., room) was displayed on the screen throughout the whole block. Participants indicated whether the target was present (by pressing P) or absent (by pressing A). The training consisted of the same visual search task performed during the testing phases and lasted 30 minutes each day. The only difference was that each participant was trained by repeating blocks in a specific target orientation (i.e., up, down, left or right) and only in a specific space (near or far). Instead, in the testing phases, participants had to complete blocks for all four orientations and in both spaces. Moreover, the training phase consisted of a total of 3,000 trials (600 each day). At the end of the experiment, participants received either credits or money as compensation for their time regardless of their performance.

### Analyses

To assess participants’ performance, their responses were divided into the proportion of hits (target present − response present) and false alarms (target absent − response present). If the participant did not press any key, the response was categorized as absent. These proportions were then used to compute the *d*-prime (*d′*) and the criterion values (Swets et al., 1961); *d′* was calculated using the formula: *d*′ = z(H) − z(FA), where z(H) and z(FA) are the *z* scores for the left-tail *p* values from the normal distribution (these can be calculated using the function “NORM.INV” in excel) for the hits and false alarms, respectively. As for the criterion, we used the following formula: c = −( z(H) + z(FA))/2. False alarms and hits proportions were adjusted to 0.01 when their values were 0, and to 0.99 when their values were 1.

Linear mixed-effects models were used to examine performance as measured by the *d*-prime (Baayen et al., 2008). Mixed-effects models include several important benefits, including the use of single-trial data rather than averaged data, the lack of an assumption of observational independence, and the inclusion of the covariance structure of the data, including random effects. A model with random effects was created. When a linear mixed model contains the most intricate random structure that does not restrict model convergence, the generalization is at its best (Matuschek et al., 2017). Following the sequential introduction of random factors, likelihood tests were used to determine how well the models fit (i.e., this was done by comparing the residuals of each model and then selecting the one with significantly lower deviance as assessed by a chi-squared test). All models had a random intercept for the participant. We started by evaluating the impact of random slopes for orientation (trained, untrained), time (before, after) and space (near, far). These were all within-participants factors. Once we found the best random slope for the model, we started to test the fixed effects. Thus, orientation, time, space, training, and their interactions were evaluated. To determine if the improvements in model fit were statistically significant, we used likelihood ratio tests as stated above (for a similar approach see also Blini et al., 2018). The raw data and the whole analysis pipeline for the linear mixed model, which was carried out with the open software R (R Core Team, 2020), are available on OSF (see link below). Such analyses have been performed on the performance in the before (i.e., Day 1) and after (i.e., Day 7) testing phases. All post hoc tests are corrected for multiple comparisons using Holm–Bonferroni.

## Results

### d prime

**Fig. 5:**
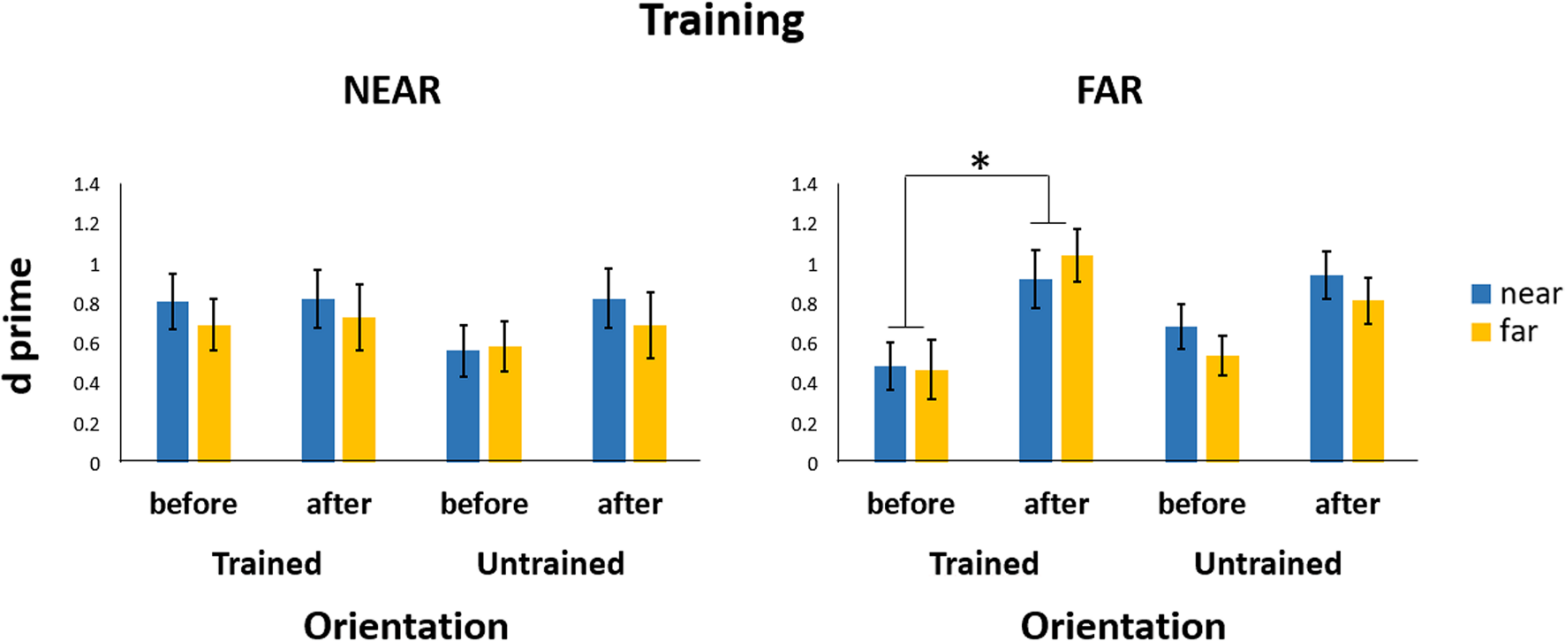
Bar charts illustrating d-prime values for the participants who trained in the near space (left panel) and those who trained in the far space (right panel). The data inside each chart are divided into Orientation (trained and untrained) and Space (near in blue and far in yellow). Error bars represent the standard error of the mean (±*SEM*). *p < .05. (Colour figure online)

Our null models contained random slopes for time when the *d* prime was evaluated. The model fit significantly improved when the model, including a main effect of time, was tested against the null model, χ^2^(1, *N* = 36) = 11.39, *p* < .001, Cohen’s *d* = 0.43, 95% CI [0.25, 0.60]. Thus, participants’ performance improved when comparing the before (*M* ± *SE* = 0.61 ± 0.08) to the after-training testing phase (*M* ± *SE* = 0.85 ± 0.09), β = 0.25, *SE* = 0.07, 95% CI [0.10, 0.38]. These results were confirmed by a two-tailed paired-sample *t* test, *t*(35) = −3.61, *p* = .001. We also found a significant two-way interaction, Training × Time, which improved the model fit, χ^2^(1, *N* = 36) = 4.72, *p* = .03, Cohen’s *d* = 0.73, 95% CI [0.46, 1.00]. Participants who carried out the training in the far space had a higher *d*-prime after (*M* ± *SE* = 0.93 ± 0.12) compared with before (*M* ± *SE* = 0.55 ± 0.11) the training β = 0.28, *SE* = 0.13, 95% CI [0.03, 0.51]. Post hoc test confirmed that there was an improvement from before to after training in the far space, *t*(34) = −4.23, *p* = .001. Finally, the Orientation × Time × Training interaction was tested against the model including their main effects and the model fit was significantly better, χ^2^(1, *N* = 36) = 13.28, *p* = .01, Cohen’s *d* = 0.88, 95% CI [0.45, 1.32]. There was a significant improvement for the participants that did the training in the far space for the trained orientation before (*M* ± *SE* = 0.48 ± 0.12) compared with after (*M* ± *SE* = 0.98 ± 0.13) the training β = −0.40, *SE* = 0.16, 95% CI [−0.70, −0.08] in both spaces. Post hoc test confirmed the results, *t*(63) = 4.72, *p* < .001. Therefore, when doing the training in the far space participants were significantly more accurate in the visual search task for the specifically trained orientation in both spaces (see Fig. 5). On the other hand, there was no significant improvement from before to after training in the near space, *t*(34) = −1.14, *p* = 0.78. Moreover, performance for the trained orientation before and after training in the near space was not significantly different, *t*(34) = −0.23,* p* = 1.00. The same was found for the untrained orientations, *t*(34) = −1.71, *p* = 0.78. Although visual inspection might suggest a positive trend for the untrained orientations after training in the far space, there was no significant post- versus pretraining improvement in performance, *t*(34) = −2.5, *p* = 0.38.

### Criterion

Our null models contained random slopes for time when the criterion was analyzed. We found a main effect of Orientation, thus the model fit significantly improved compared with the null model, χ^2^(1, *N* = 36) = 4.90, *p* = .03, Cohen’s *d* = 0.16, 95% CI [0.009, 0.30]. Participants were more conservative when tested for the trained (*M* ± *SE* = 1.18 ± 0.07) compared with untrained (*M* ± *SE* = 1.09 ± 0.07) orientations, β = −0.09, *SE* = 0.04, 95% CI [−0.17, −0.005]. The Orientation × Training interaction was tested against the model including only their main effects and fit improved significantly, χ^2^(1, *N* = 36) = 8.25, *p* = .004, Cohen’s *d* = 0.35, 95% CI [0.13, 0.56]. Participants who did the training in the near space were more conservative when tested for the trained orientation (*M* ± *SE* = 1.16 ± 0.11) compared with untrained (*M* ± *SE* = 0.96 ± 0.11), β = 0.23, *SE* = 0.08, 95% CI [0.08, 0.38].

The Orientation × Time × Training interaction was tested against the model including their main effects and the model fit was significantly better, χ^2^(1, *N* = 36) = 13.54, *p* = .009, Cohen’s *d* = 0.48, 95% CI [0.14, 0.83]. A participants’ group (i.e., near space training group) was more conservative when tested before the training for the trained (*M* ± *SE* = 1.24 ± 0.12) compared with the untrained (*M* ± *SE* = 0.95 ± 0.12) orientations, β = −0.20, *SE* = 0.16, 95% CI [−0.48, 0.10]. We also found a Training × Orientation × Space interaction, thus the model fit significantly improved compared against the null model, χ^2^(1, *N* = 36) = 12.25, *p* = .02. However, the model included only a significant Time × Training interaction, β = 0.3, *SE* = 0.19, 95% CI [0.007, 0.78]. Participants who underwent the training near were more conservative when carrying out the task in the near space for the trained (*M* ± *SE* = 1.19 ± 0.11) compared with untrained (*M* ± *SE* = 0.92 ± 0.11) orientations, Cohen’s *d* = 0.47, 95% CI [0.17, 0.77].

### RT

When response times were analyzed, the null model included random slopes for orientation and space. However, when we tested the fixed effects, the analyses revealed no significant main effects nor interactions between the variables, all χ^2^(1, *N* = 36) < 6.26, *p* > .16.

## Discussion

In the present study, we examined whether visual perceptual learning affects performance differently depending on the space in which the training is performed, namely the *peripersonal* or *extrapersonal* space, illusorily induced by leveraging the Ponzo illusion. Contrary to our predictions, based on large evidence pointing at perceptual processing advantages in the near space, we found that visual perceptual learning was effective only when participants carried out the training in the *extrapersonal* space. Moreover, when the training occurred in the *extrapersonal* space, the performance improved in both the *peripersonal* (near) and *extrapersonal* (far) spaces, showing a spatial generalization of the learning that is at odd with the largely documented specificity of perceptual learning effects, typically limited to the trained orientation, spatial location and eccentricity (Crist et al., 1997; Karni & Sagi, 1991; Schoups et al., 1995; Shiu & Pashler, 1992). Such a spatial generalization, however, emerged only for the distance component of the task as, in keeping with previous works (Sigman & Gilbert, 2000), participants were significantly more accurate in the visual search task for the specifically trained orientation in both spaces.

Typically, visual learning changes in performance are exclusive to the specific trained feature of the stimulus, and sometimes the improvement is even restricted to the trained eye and visual field, without generalization effects (Crist et al., 1997; Fiorentini & Berardi, 1980; Karni & Sagi, 1991; Schoups et al., 1995; Shiu & Pashler, 1992). However, recent studies have revealed that there are certain situations in which transfer of learning can occur (Dosher & Lu, 2017; Fahle, 2005; Sagi, 2011). Furthermore, the learning effect should not be considered completely specific, since performance on the visual search task improved substantially for the trained orientation, though a similar not significant trend was visible for the untrained orientations. Importantly, since visual search performance at baseline (i.e., before training) was not significantly different whether carried out in the near or far space, the effect we newly report here cannot be ascribed to a general difference in performance in the two spaces. Overall, these results are at odds with previous work on visual perception where an advantage for the processing of stimuli in the near compared with the far space have been recently documented (Ahsan et al., 2021, 2022; Blini et al., 2018; Dureux et al., 2021). In the following, we consider several factors that can potentially determine the unexpected far space advantage in perceptual learning.

One possibility is that our results are due to attention being deployed differentially in space during visual training. In keeping with this possibility, Abrams et al. (2008) investigated whether hand proximity alters visual processing. In their study, they used visual search, inhibition of return, and attentional blink tasks with two spatial conditions, one in which the participants’ hands were near the visual stimuli and one in which they were far from the stimuli. Similarly, to the present study, the results for the visual search task showed that participants were faster when stimuli were far from the hands. In the inhibition of return task, participants saw a peripheral cue followed by a target in the same or different locations. When the delay between the cue and the target was short, they observed a facilitation that was interpreted as an attentional engagement at the cued location (thus shorter RT), whereas at long delays there was a slower response that they interpreted as an inhibition due to attentional disengagement and the return of attention to the cued location (longer RT). They found lower inhibition of return only when stimuli were presented near the hands, whereby participants’ attentional disengagement from the cued location was delayed/disrupted. In their last task, participants had to detect two targets amongst a series of stimuli presented rapidly. When the time between the two targets was around a few hundred milliseconds, the identification of the second target was impaired (attentional blink). The results demonstrated higher difficulty in attentional disengagement from the first target when hands were near compared with far from the stimuli. Based on these findings, in our study participants might have had more difficulties in moving their attention rapidly and between objects (triangles) in the near space. Even though no difference between spaces was evident at baseline, attentional processes might have implied at larger extent during the perceptual training in the extrapersonal space, where participants could more easily disengage and reorient attention.

Additionally, the Ponzo illusion is an optical illusion that, due to the perspective cues, leads to perceiving stimuli that appear further in space as larger compared with stimuli in the near space even though they have the same physical size and identical retinal projection (Gillam, 1973; Gregory, 1963; Leibowitz et al., 1969; Prinzmetal et al., 2001). Thus, participants were likely to perceive stimuli in the far space as relatively larger and this might have facilitated visual learning in the far space. According to this view, the illusion might have led to higher visual acuity in the far space due to size-constancy mechanisms (Kersten & Murray, 2010). In this regard, it has been shown that orientation discrimination (Schindel & Arnold, 2010) and letter recognition (Lages et al., 2017) improve if the pattern appears larger. In our study, such effect may have enhanced selectively the learning phase, though not the perceptual processing in that space. Indeed, before the training participants’ performance when the target was in the illusory far compared with the near space (i.e., illusory bigger size) was comparable. However, stimuli in the near space should still have been perceived similar in size compared with those originally used by Sigman and Gilbert (2000). In this respect, here we should have observed performance improvement similar to their work, though this was not the case. This suggests that, when perceptual learning is engaged across space in depth, far(ther) distances may benefit from the most, if not all the training induced improvement. Thus, the mere presence of the two spaces (near and far), namely depth perspective, may not be directly comparable with the situation in which only one space is present as in Sigman and Gilbert (2000) study.

Another, non-mutually exclusive possibility is that the greater effectiveness of visual learning in the far compared with the near space could be originating from an evolutionary mechanism. Previc (1990) theorized an ecological perspective of the functional segregation of the near and far visual processing, which appears to have a bias towards the lower and upper visual fields, respectively. The critical link between near space (peripersonal space) and visuomotor skills, as well as the far space (extrapersonal space) and visual searching abilities, can be traced back to the change to an erected position (Allman, 1977; Bishop, 1962; Goldman-Rakic, 1987; Hewes, 1961; Hunt, 1994; Polyak, 1957; Richmond et al., 2001; Snodderly, 1979; Will, 1972). Recent studies (Nasr & Tootell, 2018, 2020) have shown that in the brain areas involved in visual depth perception (V2, V3A), neurons representing the lower visual field respond more strongly to near compared with far stimuli, whereas neurons that represent the upper visual field have the opposite pattern. These results are compatible with the idea of different stimulus processing by the visual system for the lower-near and upper-far visual fields based on ecological (see above) and statistical frequencies of natural environments (Yang & Purves, 2003). Moreover, Nasr and Tootell (2018, 2020) also found that in V3A there are “near-preferring” clusters of neurons that had a bias toward low spatial frequency (global) visual perception compared with far-preferring ones. Both vision and hearing appear to be related to the statistics of the environment. Specifically, sounds at higher frequencies tend to be at an elevated point (head-centred), thus there is a frequency-elevation mapping, and the outer ear anatomical conformation seems to maximize this mapping (Parise et al., 2014). Visual stimuli in natural scenes tend to be further when they are in the upper visual field and closer when they are in the lower visual field (Ooi et al., 2001; Yang & Purves, 2003). Furthermore, Blini et al. (2018) used a 2D illusory setting similar to the one used in the present work, and reported that monocular depth cues were sufficient to segregate EPS from PPS, as they found a PPS advantage in a Ponzo-like display comparable to that observed in their 3D setting (virtual reality). While future studies would benefit from estimating the illusory perceived distances, we infer that the 2D setting used in the present study was also adequate to segregate near and far spaces. In sum, although in our experiment the predisposition to visual search in far space and upper visual field could not be observed before the training, it might have become relevant during the training/learning phase.

Although it is out of the scope of this study to discern the relative contribution of the factors considered above, they seemingly point to a common feature we could term as a “predisposition” to visual perceptual learning for stimuli that are visually far (or illusorily perceived as such). Moreover, we would like to note the possibility that visual perceptual learning for near stimuli may require longer training compared with far stimuli, as suggested by the fact that performing the training in near space did not improve visual search of the trained orientation. Importantly, none of the previous research investigating the difference in peripersonal and extrapersonal space examined the effects of training. Thus, the present findings pave the way for new research avenues toward the relationships between perceptual learning and perceived distance.

## Data Availability

All data have been made publicly available via OSF (https://osf.io/9a4x8/?view_only=5e773984ee8549b3ac4404e1c7ba0543). The design and analysis plan for the experiment were not preregistered.
